# Nanoparticle-based CT visualization of pulmonary vasculature for minimally-invasive thoracic surgery planning

**DOI:** 10.1371/journal.pone.0209501

**Published:** 2019-01-17

**Authors:** Hsin-pei Hu, Harley Chan, Hideki Ujiie, Nicholas Bernards, Kosuke Fujino, Jonathan C. Irish, Jinzi Zheng, Kazuhiro Yasufuku

**Affiliations:** 1 Division of Thoracic Surgery, Toronto General Hospital, University Health Network, Toronto, Ontario, Canada; 2 TECHNA Institute, University Health Network, Toronto, Ontario, Canada; 3 Institute of Biomaterials & Biomedical Engineering, University of Toronto, Toronto, Ontario, Canada; University of Missouri Columbia, UNITED STATES

## Abstract

**Purpose:**

To evaluate CF800, a novel lipid-based liposomal nanoparticle that co-encapsulates indocyanine green (ICG) and iohexol, for CT imaging of pulmonary vasculature in minimally-invasive thoracic surgery planning.

**Methods:**

CF800 was intravenously administered to 7 healthy rabbits. *In vivo* CT imaging was performed 15 min post-injection, with a subset of animals imaged at 24h, 48h, and 72h post injection. Signal-to-background ratios (SBR) were calculated at the inferior vena cava and compared across time-points. A similar protocol was applied to 2 healthy pigs to evaluate the feasibility and efficacy in a large animal model. To evaluate the feasibility of clinical application, a survey was completed by 7 surgical trainees to assess pre- and post-injection CT images of rabbits and pigs. Responses on the discernibility of pulmonary vasculature sub-branches and comfort level to use the images for pre-operative planning were collected and analyzed.

**Results:**

CF800 injection improved visualization of pulmonary vessels in both rabbit and pig models. The SBR of rabbit pulmonary vasculature was significantly higher after CF800 injection (range 3.7–4.4) compared to pre-injection (range 3.3–3.8, n = 7; p<0.05). SBR remained significantly different up to 24 hours after injection (range 3.7–4.3, n = 4; p<0.05). Trainees’ evaluation found the post-injection CT images had significantly higher discernibility at the second vessel branch generation in both rabbit and pig models. Trainees identified smaller vasculature branch generations in the post-injection images compared to the pre-treatment images in both rabbit (mean 6.7±1.8 vs 5.4±2.1; p<0.05) and pig (mean 6.7±1.8 vs 5.4±2.1; p<0.05). Trainees were significantly more comfortable using post-injection images for surgical planning compared to the pre-injection images (rabbit: 8.1±1.1 vs. 4.7±2.1; pig: 7.6±2.1 vs. 4.9±2.2; p<0.05).

**Conclusion:**

CF800 provides SBR and contrast enhancement of pulmonary vasculature which may assist in pre-surgical CT planning of minimally invasive thoracic surgery.

## Introduction

Lung cancer is the leading cause of cancer death worldwide, accounting for 1.69 million deaths in 2015 [1]. The detection of patients with early-stage lung cancer is expected to increase as computed tomography (CT) technology improves and more low-dose CT screening programs are implemented to leverage the mortality reduction benefits of early screening [2]. Minimally invasive surgical (MIS) techniques such as video-assisted thoracic surgery (VATS) have become the standard of care for surgical resection of early-stage lung cancers. Since the randomized controlled trial reported by Lung Cancer Study Group, lobectomy has been established as the gold standard surgical approach for early-stage lung cancer due to higher survival rate and lower recurrence rates compared to sublobar resection [3].

There is ongoing debate whether segmentectomy would an acceptable alternative to lobectomy for stage I non-small cell lung cancer (NSCLC) [4]. Segmentectomy may be a feasible alternative to lobectomy for selected patients who could tolerate either procedure [5]. Retrospective studies show segmentectomy and lobectomy may have similar survival rates and recurrence rates in patients with stage IA NSCLC of up to 2 cm tumor size with ground glass features [6–10]. Though the postoperative functional benefit of segmentectomy over lobectomy is contested [11,12], segmentectomy is a reasonable lung parenchyma-sparing alternative for appropriately selected patients with limited cardiorespiratory reserve [13–16]. Segmentectomy may be the preferred curative procedure for surgical candidates with multiple synchronous NSCLC located in multiple lobes to be resected [17]. However, segmentectomy is more technically challenging than lobectomy as it requires identification of complex anatomical references for proper planning and resection [18]. Technical and anatomical difficulties of segmentectomy can contribute to higher rates of positive surgical margins and inadequate lymphadenectomy, potentially leading to higher locoregional recurrence and lower overall survival compared with lobectomy [19–21]. Additionally, anatomic variations of pulmonary vessels are common and can increase risks of injury in lung resections [22]. The major vessels and branches of the pulmonary artery are the most frequent sites of intraoperative vessel injury in VATS [23]. While the overall incidence of bleeding complications during or after VATS is rare, bleeding from one of the main pulmonary vessels is a serious complication in VATS anatomic resections of early-stage lung cancer that may require conversion to thoracotomy [24,25]. Proper preoperative surgical planning with review of the surgical anatomy can lead to safer and more effective segmentectomy procedures for treating early stage NSCLC [26].

Preoperative surgical planning can be achieved using three-dimensional computed tomography (3D-CT) and angiography to visualize pulmonary vasculature [27–29]. The three-dimensional images are processed and viewed on a workstation to identify target vessels and bronchi, anatomical variations, and lung nodule location prior to the surgery. After segmenting relevant anatomical structures, the volume-rendered structures of interest can be viewed prior to or during the surgery. This method can help preoperative identification of pulmonary vessel branches larger than 1.5 mm for safer anatomic pulmonary resections [27,28]. However, the procedure is highly time-sensitive as the CT scan needs to be performed immediately after contrast medium injection. While the preoperative images can be viewed intraoperatively on monitors, anatomical structures are manipulated and displaced such that the surgeon’s familiarity of the pulmonary vessels configuration is still heavily relied upon. Thus, true intraoperative localization of the vasculature during VATS segmentectomy currently still relies on thoracoscopic visualization.

CF800 is a novel lipid-based liposomal nanoparticle that co-encapsulates indocyanine green (ICG) for near infrared fluorescence (NIR) imaging and iohexol for CT imaging [30–32]. This nanoparticle, developed by Zheng *et al*, is a potentially powerful dual-modality imaging agent that allows for longitudinal pre- and intraoperative imaging via NIR fluorescence and CT. The unique size and particle characteristics of the PEGylated liposome minimizes uptake by the mononuclear phagocytic system, allowing the particle to be retained in circulation and at sites of enhanced vascular permeability [31]. As tumor sites have increased vessel permeability that enables macromolecular drug accumulation via the enhanced permeability and retention (EPR) effect [33], CF800 can be used for contrast-enhanced pre- and intraoperative imaging and localization of tumors. Investigation in rabbit lung and head & neck cancer models have shown effective accumulation and visualization of the imaging agent in solid tumors [31,34]. CF800 used in conjunction with CBCT guidance has the potential for intraoperative, real-time tracking of pulmonary vasculature and tumor.

The goal of this study was to apply cone-beam computed tomography (CBCT) scanning technology to healthy animal models for evaluation of the potential clinical benefits of CF800 to enhance CT visualization of pulmonary vasculature. CBCT has a mobile gantry capable of fast acquisition speeds, fast processing time for 3D images, sub-millimeter spatial resolution, and lower radiation dose compared to conventional CT [35]. Co-encapsulated with ICG for NIR imaging and iohexol for CT imaging, CF800 enables NIR fluorescence-based visualization and localization of lung tumors in mice and rabbit models [32,36]. This study evaluates the CT contrast enhancement of CF800 and its effect on assisting CT-based clinical evaluation and surgery planning using healthy rabbit and pig models.

## Materials and methods

### CF800 formulation

The CF800 liposome imaging agent infused during the imaging procedure was prepared and characterized according to a previously published method [32]. Briefly, the CF800 liposome bilayer is composed of 1,2-dipalmitoyl-sn-glycero-3-phosphocholine (DPPC, CordenPharma, Liestal, Switzerland), cholesterol (Northern Lipids Inc., Vancouver, BC, Canada) and 1,2-distearoyl-sn-glycero-3-phosphoethanolamine-N-[poly(ethylene glycol)2000] (PEG2000DSPE, CordenPharma, Liestal, Switzerland) in 55:40:5% mole ratios. The liposome co-encapsulates ICG (IR-125, Acros Organics, Geel, Belgium) and the CT contrast agent Omnipaque350 (Nycomed Imaging AS, Oslo, Norway). The liposome mixture was first dissolved in ethanol and then hydrated for 4 h at 70°C in a solution of 300 μg of ICG dissolved in Omnipaque350. The resulting solution was extruded at 100 to 400 psi pressure using the *LIPEX* Extruder (Northern Lipids Inc., Vancouver, Canada). Purification was performed with a Sephadex G-25 column.

The size of the liposome samples was measured using dynamic light scattering (90Plus, Brookhaven, Holtsville, New York). The iodine and ICG concentrations were determined using an absorbance assay at a wavelength of 245 nm for iodine and 785 nm for ICG (Cary 50 UV/VIS Spectrophotometer, Varian Inc., Palo Alto, California). The average size of CF800 used in this study was 94.6±2.6 nm from 5 different production batches. The average ICG and iodine concentration was 125.6±13.3 μg/mL and 51.8±1.9 mg/mL, respectively.

### Animal models

The animal experiments protocols in this study were approved by the Toronto General and Western Animal Care Committee. All procedures including injection of CF800 and CBCT scanning were conducted under anesthesia with isoflurane inhalation gas: 5% for induction and 2% for maintenance. Appropriate depth of anesthesia was evaluated with criteria including muscle relaxation, toe pinch, jaw tone, respiration pattern, and color of mucous membranes. Each animal received CF800 formulation in a slow bolus (0.5 mL/s) and CT imaging was acquired (Siemens *PowerMobil* [37]). During the recovery period, animals were placed on a heating pad, supplemental oxygen was administered, and animals were monitored every 10 minutes until they were responsive and able to intake food and water. Animals were sacrificed in accordance with University Health Network Humane Endpoint Guidelines, if humane endpoints were reached such as lethargy, weight loss exceeding 20% of body weight, dyspnea, inadequate food or water intake, or inability to ambulate. Two rabbits died from respiratory distress while recovering from breath-hold during the CT imaging procedure (see below). The breath-hold induction and recovery protocols were further optimized thereafter. Euthanasia was achieved by rapid infusion of potassium chloride while under anesthesia with isoflurane gas.

### CT imaging in rabbits

General anesthesia was induced and maintained in healthy rabbits (~3 kg) with inhaled isoflurane (2–5%) in an animal operating room. A laryngeal mask was placed, and the rabbit was secured in the prone position within the CBCT scan area. The following CBCT scanner settings were used: 100 kVp, 2.6 mA, 200 projections for ~178°, 1 min rotation time, and 500 x 500 x 384 voxels at 0.4 mm voxel size with a field of view of 20 cm x 20 cm x 15 cm at the isocenter. Rocuronium bromide (0.08 mg/kg IV) was used to induce temporary breath-hold during CT scans to eliminate motion artefacts. An initial pre-injection CT scan was acquired. Then, 20–25 mL of CF800 formulation was infused intravenously through a catheter in the ear. A second CT scan was performed 5 minutes post injection. CT scans were repeated at 24, 48, and 72 hours post injection.

### CT imaging in pigs

Healthy male *Yorkshire* pigs (~30 kg) were sedated with ketamine (20 mg/kg IM), midazolam (0.3 mg/kg IM), and atropine (0.04 mg/kg IM) before being transferred to the animal operating room. General anesthesia was then induced with 5% isoflurane inhalation gas through a breathing mask and maintained at 2% isoflurane. Additional propofol (5 mg/kg/h of IV) was administered if needed to achieve an appropriate depth of anesthesia. Tracheostomy was performed to intubate the animal with an endotracheal tube. The pig was then positioned on the surgical table in the left or right lateral decubitus position. The CBCT was then positioned over the thorax for image acquisition, using the scanner settings as above. Rocuronium bromide (0.4 mg/kg IV) was infused prior to the CT scan to induce breath-hold and reduce breathing motion artifacts. An initial CT scan was acquired. Then, dexamethasone (2.5 mg/kg IV) was infused, followed by 200 mL of CF800 formulation through a catheter in the ear. A second CBCT scan was acquired 5 minutes post injection before sacrificing the animal.

### Image analysis

The CT images were segmented and analyzed using ITK-SNAP software [38]. The volumes-of-interest (VOIs) were segmented using semi-automated threshold-based active contouring. Segmented blood vessels included the aorta, inferior vena cava, pulmonary artery, and pulmonary veins. Background included the air in the trachea from pharynx to carina. The mean and standard deviation of the CT attenuation was calculated for each VOI. Signal-to-background ratio was calculated as the ratio between the mean signal intensity of the inferior vena cava and the mean signal intensity of the tracheal air of each animal.

### Evaluating the potential application of CF800 in thoracic surgery

A survey was distributed to thoracic surgeons and general surgeons-in-training to evaluate the potential clinical application of CF800 in planning pulmonary surgical resections. Participants reviewed a set of CT images of rabbit and pig: one acquired at pre-injection and one at post-injection of CF800. The participants were blinded on whether the image was acquired before or after CF800 injection. The CT images of the same animal subject were shown and the participants can freely change the windowing and level settings. Participants were asked to distinguish the aorta, inferior vena cava, and pulmonary arteries down to the second branch generation on each image set, rating the discernibility of each blood vessel branch on a 5-point scale. They were also asked to identify the smallest discernable vessel branch generation. Participants rated their comfort level of using the shown CT image set for surgical planning on a 10-point scale. They were then asked whether they could identify which CT image sets had contrast enhancement.

### Statistical analysis

The data analysis for this investigation was generated using the Real Statistics Resource Pack software (Release 5.4) [39]. The signal-to-background ratios at pre- and post-injection of CF800 were compared using two-tailed student’s T-test with 95% confidence interval. The survey results were analyzed using Wilcoxon Signed-Ranks Test for paired samples, where p < 0.05 is deemed to be statistically significant.

## Results

### Visualization of rabbit pulmonary vasculature with CF800

**Fig 1:**
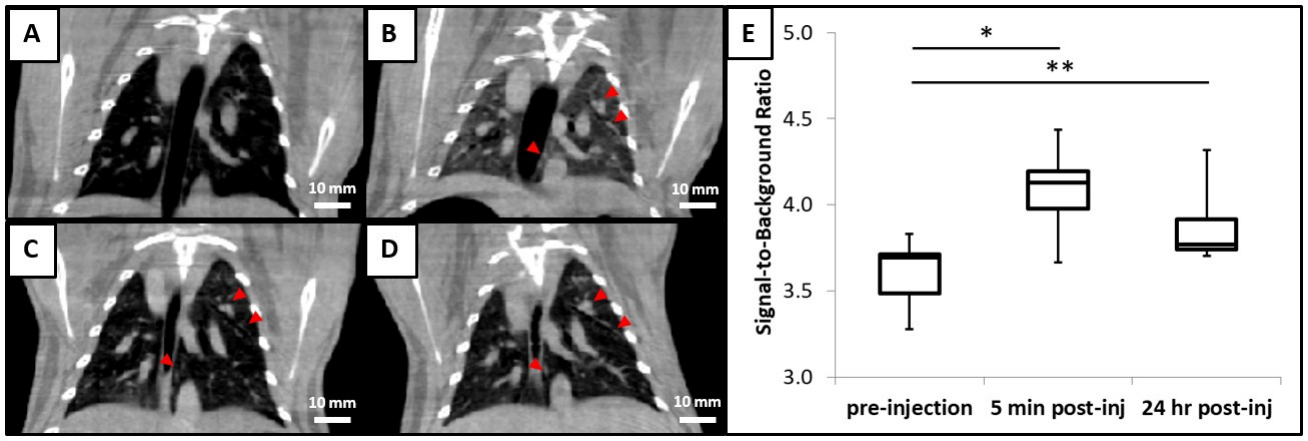
Rabbit inferior vena cava on cone-beam CT images has significant increase in signal-to-background ratio for up to 24 hours after CF800 injection. (A)-(D): Representative cone-beam CT images acquired at different time points: (A) Before CF800 injection; (B) 5 minutes post-injection; (C) 24 hours post-injection; and (D) 72 hours post-injection. Enhanced contrast of pulmonary vasculature can be seen immediately following CF800 injection and persists up to 72 hours (arrowheads). Images are displayed at the same window plane and intensity level (scale = 10 mm). (E) SBR is significantly increased 5 minutes after CF800 injection in paired t-test analysis (n = 7; *p = 3x10^5) and up to 24 hours after CF800 injection (n = 4; **p = 0.02).

**Fig 2:**
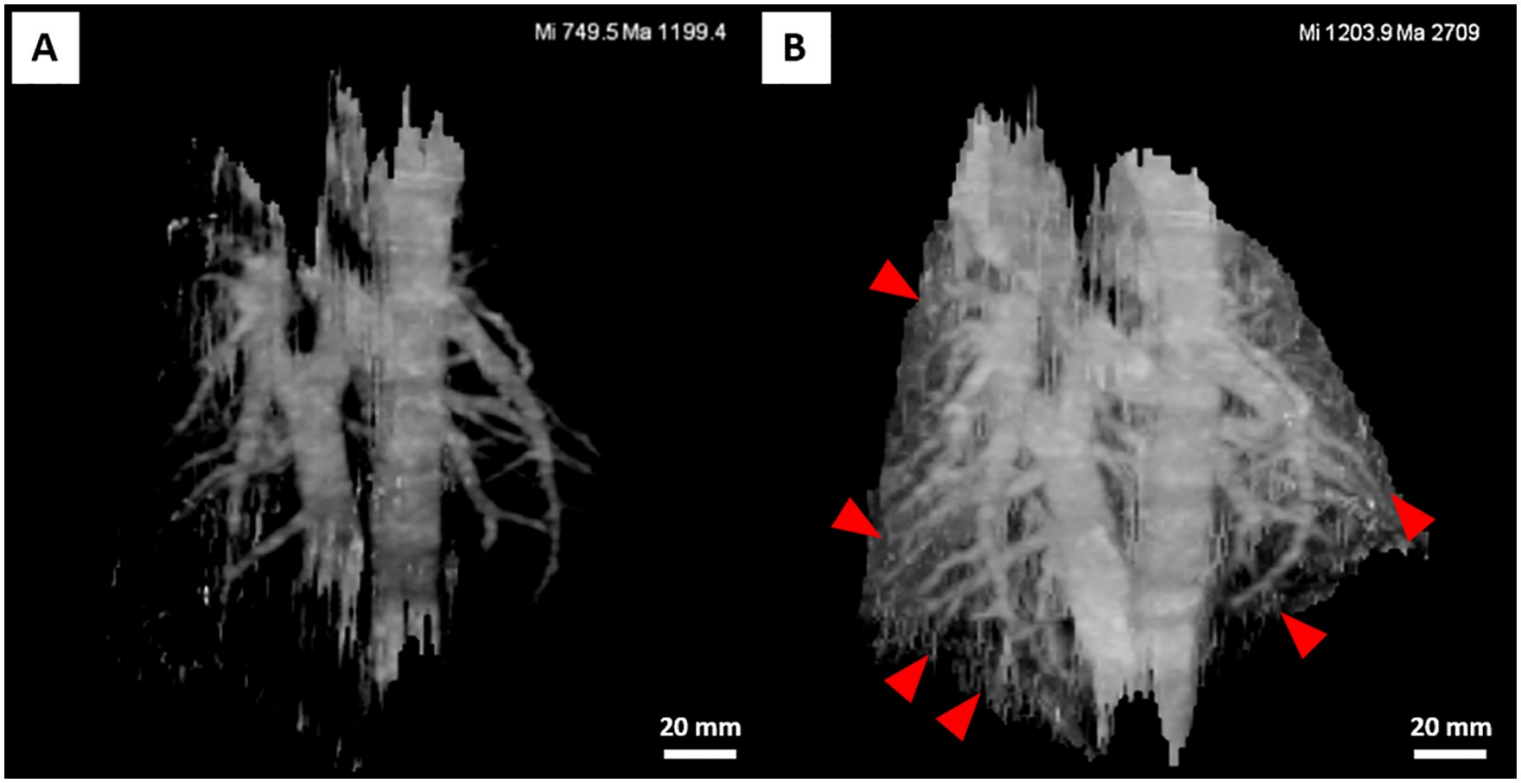
Representative 3D reconstruction of rabbit pulmonary vasculature shows increased vascular branch visualization following CF800 injection. (A) Pulmonary vasculature in the CT image acquired before CF800 injection. (B) Pulmonary vasculature in the CT image acquired 5 minutes after CF800 injection. Images are depicted using a thresholding-based method with lower and upper threshold values set at 25–40% of maximum intensity within the segmented lungs (values on top-right). Small pulmonary vasculature branches at the distal branch generations can be segmented in the post-injection image due to increased attenuation (arrowheads).

CBCT images of rabbits were acquired before and 5 minutes, 24 hours, 48 hours, and 72 hours after CF800 injection (Fig 1). The pulmonary vasculature can be identified in the CBCT images prior to CF800 injection (Fig 1A). Immediately after the injection, there was an increase in attenuation within the lung volume (Fig 1B). However, this diffuse increase in attenuation cleared in subsequent time-points, leaving only the pulmonary vasculature attenuation enhanced (Fig 1C and 1D). A greater number of distinguishable small pulmonary vessel branches was observed in the post-injection images compared to pre-injection. 3D reconstruction of the segmented pulmonary vasculature further illustrated the increase in distinguishable pulmonary vessel branch generations after CF800 injection (Fig 2).

After CT image segmentation, the signal-to-background ratio (SBR) of rabbit pulmonary vasculature was calculated from the attenuation of inferior vena cava relative to tracheal air. As expected, the SBR in the post-CF800 injection images (mean 4.1; range 3.7–4.4; n = 7) were significantly higher than SBR in the pre-injection images (mean 3.6; range 3.3–3.8; n = 7) (Fig 1E). Applying the same quantitative analysis method for images acquired at multiple time points, the difference in SBR remained significant at 24 hours after CF800 injection (mean 3.9; range 3.7–4.3; n = 4).

### Visualization of porcine pulmonary vasculature with CF800

**Fig 3:**
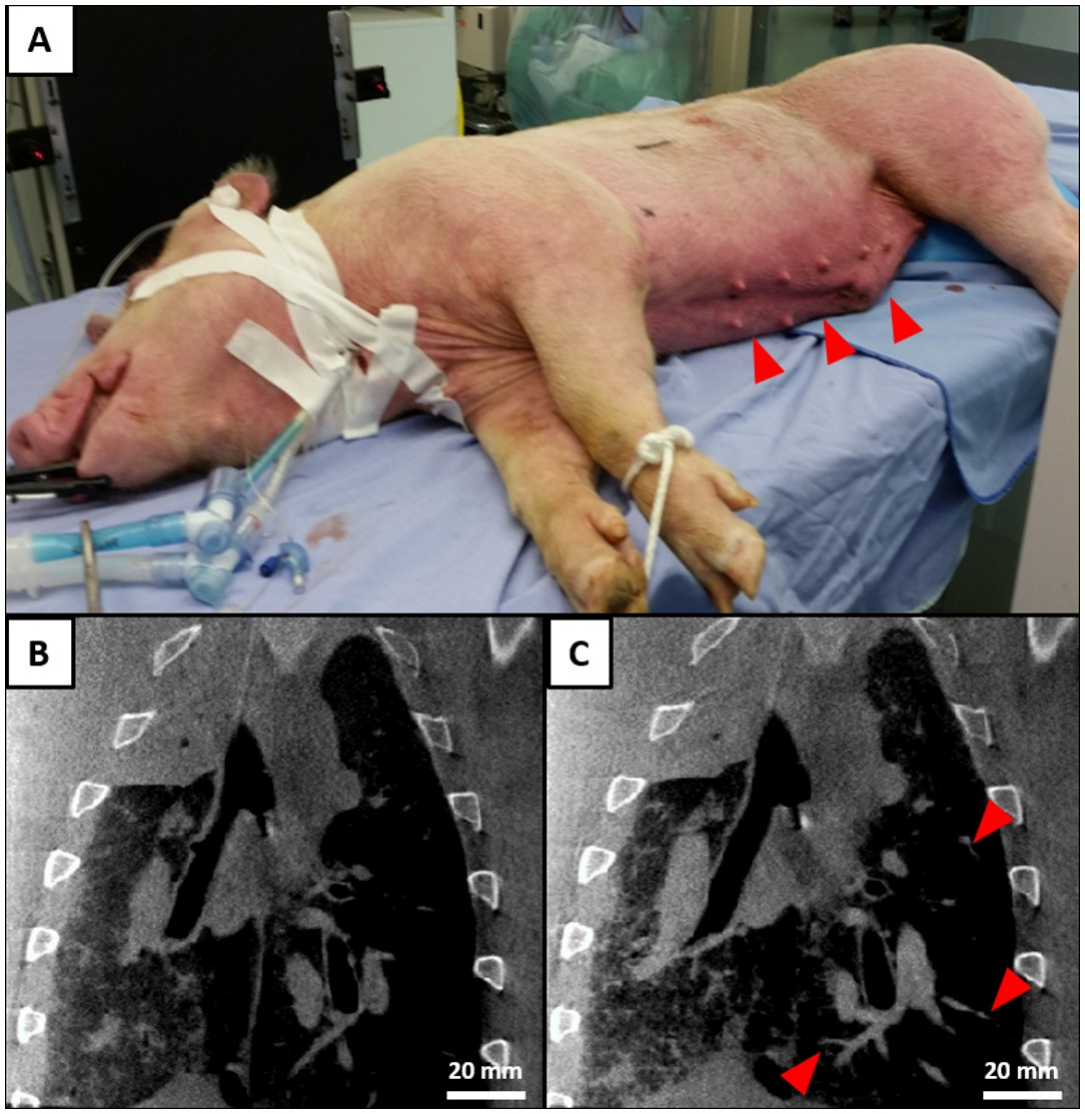
CBCT scanning procedure for porcine model and representative cone-beam CT coronal images for porcine lungs (n = 2). (A) CBCT images were acquired in the lateral decubitus position. Transient localized erythema (arrowheads) following an initial injection of 30 mL CF800 spontaneously resolved 15 minutes after the initial hypersensitivity reaction. CBCT coronal sections acquired at different time points: (B) before CF800 injection; (C) 15 minutes post-CF800 injection. Increased visualization of distal vasculature branches can be observed in the post-CF800 injection image (arrowheads). Images are displayed at the same windowing and level (scale = 20 mm).

CBCT images were acquired to visualize the pulmonary vasculature in two pigs. After CF800 injection, the animals developed transient regional erythema and tachycardia, consistent with complement activation-related pseudoallergy (CARPA) reaction described in literature (Fig 3A). Similar to the rabbit model, CBCT images acquired after CF800 injection demonstrated improved visualization of smaller pulmonary vasculature branches compared to pre-treatment (Fig 3B and 3C).

### Survey evaluation of clinical application of CF800

**Fig 4:**
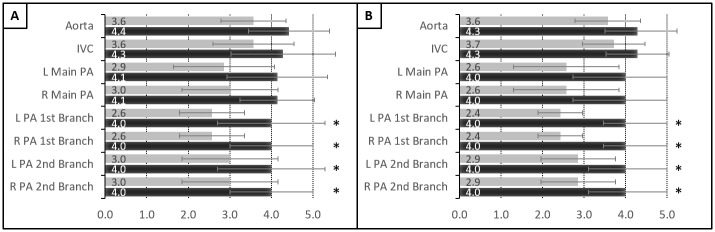
Trainees’ subjective rating on the discernibility of pulmonary vasculature on CT images before and after CF800 injection. (A) Rabbit (B) Pig (Scale 1–5; Mean±SD; *p < 0.05). Legend—grey: before CF800 injection; black: after CF800 injection.

Six thoracic surgery fellows and one general surgery resident (average number of post-graduate years: 8.9 (range 2 to 14)) were surveyed to evaluate the quality of CT images acquired in rabbit and pig at pre-injection and post-injection of CF800. Surveyees were asked to rate the discernibility of pulmonary vasculature on a scale of 1–5 at each branch generation (Fig 4). There was no statistically significant difference in the visualization of the aorta, IVC, and pulmonary artery main branch between pre- and post-injection images (p = 0.07–0.11). However, discernibility was significantly different at the first and second branch generations of the pulmonary arteries in both rabbit (Fig 4A; p<0.05) and pig (Fig 4B; p<0.05).

**Fig 5:**
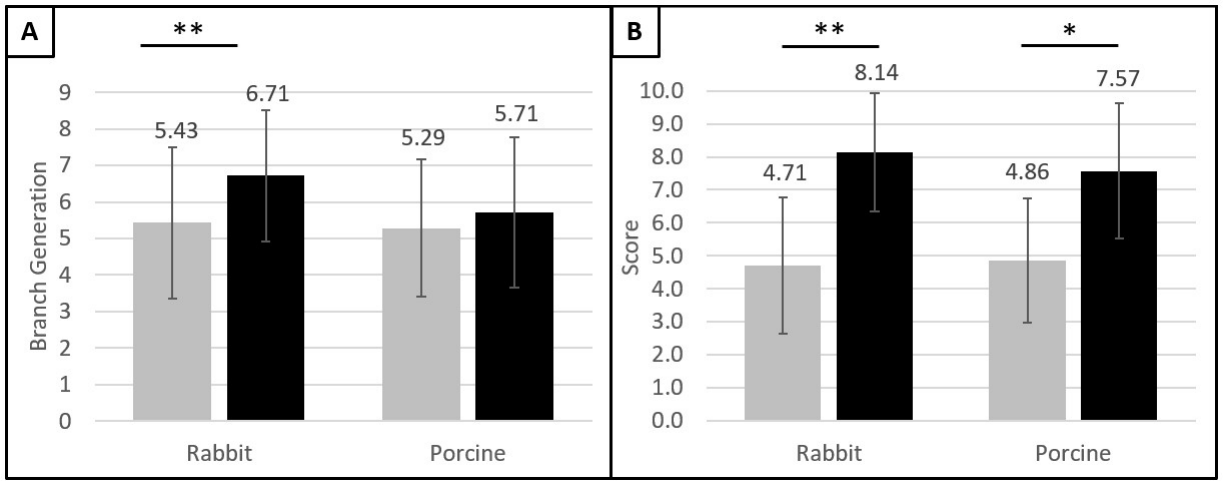
Trainees’ subjective ratings on pre- and post-injection images. (A) Smallest discernable pulmonary vessel branch generation; (B) Comfort level in performing a lobectomy with the images (Mean±SD; *p < 0.05, **p < 0.005). Legend—grey: before CF800 injection; black: after CF800 injection.

In the evaluation of clinical applicability, the smallest pulmonary vessel branch generation that the trainees could identify with confidence in rabbit CT images was significantly increased in the post-injection images (mean 6.7±1.8) compared to the pre-injection images (mean 5.4±2.1; p<0.05) (Fig 5A; Table A in S1 Text). In the pig CT images, the smallest identifiable pulmonary vessel branch generation did not differ significantly between post-injection (mean 5.7±2.1) and pre-injection (mean 5.3±1.9; p = 0.29). For comfort level in using the CT images for pre-operative planning, trainees were significantly more comfortable using the post-injection images (rabbit: mean 8.1±1.1; pig: mean 7.6±2.1) compared to the pre-injection images (rabbit: 4.7±2.1; pig: 4.9±2.2; p<0.05) (Fig 5B; Table B in S1 Text). All the trainees correctly identified the post-injection images in the CT image pairs when surveyed afterwards.

## Discussion

Identifying anatomical references for resection is essential for safe VATS segmentectomy of lung nodules [18]. This study demonstrates systemic administration of CF800 can improve CT-based surgical planning by enhancing visualization of pulmonary vasculature. Previous studies demonstrated the localization of CF800 to malignant tissue enables lung tumor identification and delineation with real-time intraoperative NIR fluorescence-based imaging [32,36]. This study shows the additional benefits of using CF800 administration to increase vessel discernibility beyond the first pulmonary artery branch generation and to potentially increase surgeons’ comfort level in performing pulmonary resections.

CF800 was the first reported dual modality CT and NIR visualization agent specifically designed for image-guided surgery applications [32]. Sheng et al. developed a nano-liposome (LIP-PFOB-ICG) that co-encapsulates perfluorooctyl bromide (PFOB) and ICG for multimodal imaging [40]. Sheng et al. demonstrated in mice bearing subcutaneous MDA-MB-231 tumors that LIP-PFOB-ICG passively accumulates in the tumors via EPR effect. LIP-PFOB-ICG potentially enables photoacoustic imaging and image-guided phototherapy, but it is less unsuitable for pulmonary vasculature visualization in surgical planning as it has a short vascular circulation half-life (3.32 hrs) compared to CF800 (~25 hrs) [32]. Characterization of LIP-PFOB-ICG in larger animal models is currently pending formal investigation.

CF800 has stable co-encapsulation of iohexol and ICG in liposomes that conserves their imaging properties for several days. Previous pharmacokinetic evaluation of CF800 in a rabbit model demonstrated persistent vascular enhancement by greater than 200 Hounsfield Units for up to 24 hours [32]. This study confirms the significant increase of signal-to-background ratio for circulating blood immediately after CF800 injection and after 24 h in the rabbit study model. The accumulation of CF800 in tumors also peaks after 24 hours and is retained for up to 96 hours due to the EPR effect at the nearby vessels with increased permeability [41]. Thus with this strong uptake and subsequent retention profile, CF800 has high potential utility in both pulmonary vasculature and lung tumor localization during VATS anatomic resection [31,32,34].

Survey participants identified more pulmonary vessel branch generations in the post-injection image of the rabbit model, suggesting visualization of smaller distal branches is enhanced. The limits of discernibility appear to be at the 9^th^ generation, likely a combination of factors including smaller vessel size along with higher background by the supernumerary vessels of the same or higher generation (Table A in S1 Text). Interestingly, survey participants did not identify more branches in the porcine model. One potential reason is the presence of more CT artifacts due to the animal’s denser tissue and bony structures. Beam hardening artifacts more frequently occur with radio-dense objects as they more rapidly absorb the lower-energy photons within a polychromatic X-ray beam. These can be streaking artifacts (multiple dark streaking bands between two dense objects) or cupping artifacts (false brightness along the periphery of an object) [42]. While CT scanner calibration reduces these artifacts, unavoidable beam-hardening artifacts will have more effect on the CBCT image quality for the pig models compared to rabbit. Another potential cause to the discrepancy between animal models is related to the lumen diameter relative to vessel wall. The smooth muscle thickness is 2.0–6.9% of the external diameter of the pulmonary artery in humans, 12.3–12.8% in rabbits, but up to 17.0–23.9% in pigs [43]. Contrast enhancement of circulating blood would be more observable in humans and rabbits compared to pigs due to the larger proportion of the lumen. Nonetheless, the blinded surveyees felt more comfortable working with the post-injection images for surgical planning, indicating identification of higher branch generations may not be essential to the potential clinical utility of CF800.

This study marks the first large animal investigation of the CF800 nano-liposome. Domestic pigs are suitable animal model to translate nano-liposome technology to humans due to the similar anatomy, physiology, and functional immune system [44–46]. This pilot investigation demonstrated the safety and feasibility of formally evaluating the biodistribution and pharmacokinetics of CF800 in pigs in future studies. Pigs are known to be extremely sensitive to complement activation-related pseudoallergy (CARPA)–a stress reaction common in nanomedicines with PEGylated surfaces [46–48]. The pigs used in this study demonstrated transient reactions consistent with CARPA. This sensitivity is noted to be greater than that seen in humans. Studies with other formulations suggest liposomes can trigger complement activation due to their similar size and shape to pathogenic microbes, as well as lack of complement control proteins on the particles surface [49]. This hypersensitivity reaction manifests as cardiovascular, hematological, and skin changes and can rarely cause severe cardiopulmonary symptoms [50,51]. The general approach to preventing and reducing CARPA include administering antihistamines and corticosteroids, as well as reducing the rate of infusion [52,53]. In this study, these measures eliminated the CARPA reaction within fifteen minutes of onset, suggesting this transient hypersensitivity reaction can be well-controlled with proper preparation. Doxil, an FDA-approved PEGylated doxorubicin HCl nano-liposomal drug, provides a strong foundation to overcoming CARPA reactions in future clinical use of CF800 nano-liposomes as it similarly induces CARPA as a potential side effect [54].

Injection of contrast to improve resection planning is not a novel concept. Studies have used multidimensional CT imaging along with injected iodinated contrast medium to render the pulmonary arteriovenous structures in 3D prior to the surgical procedure [28,55–58]. Preoperative CT-based planning offers similar operative time, mortality, morbidity, and complication rates with the advantage of identifying difficult surgical cases early [55]. Other studies have demonstrated intraoperative identification of intersegmental planes after segmental artery ligation and distal injection of ICG or indigo carmine dyes [59–63]. This approach allows identification of resection planes by naked eye or with NIR fluorescence, potentially enabling safer and effective resection. A single preoperative administration of CF800 enables both enhanced CT-based surgical planning and intraoperative NIR guidance, making it a potentially powerful addition to VATS.

The use of CBCT in this study is motivated by the eventual clinical application of CF800 in an intraoperative setting. CBCT images provide sufficient contrast-to-noise ratio for visualizing lung nodules, allowing intraoperative CBCT techniques such as CBCT-guided lung biopsy to be developed [64]. Intraoperative CBCT imaging combined with navigation is an emerging technology in oncologic surgery, providing increased accuracy and confidence, and decreased task workload [65–67]. CBCT can be integrated with an optical tracking system and endoscopic video to enable high-precision, real-time tracking of anatomical structures and surgical instruments [37]. In such applications, regions of interest can be segmented, registered and displayed as an overlay to the real-time endoscopic image during surgery, providing a powerful intraoperative tool for visualization. Once the systemic administration of CF800 is integrated to the surgical workflow, the persistent contrast enhancement of CF800 would help localize and delineate pulmonary vasculature and target tumor as part of an intraoperative CT imaging technique for definitive anatomic resections of lung lesions.

Limitations of this study pertain to the qualitative survey used to collect clinically-oriented application of the nano-liposome. There was a risk of response bias since the respondents were able to correctly identify whether the shown CT images was acquired before or after CF800 injection despite randomization and blinding steps. While pig and human lungs share similar dimensions, respondents were required to speculate the clinical application of the nano-particle while examining images of non-human subjects. Another limitation is the limited number of pig models used in this pilot investigation. Nonetheless, this study offers an encouraging outlook at the potential role of CF800 in planning minimally-invasive lung cancer surgeries. Increasing the sample size in future studies will enable quantitative analysis and formal characterization of CF800 in the porcine model, including its biodistribution profile, clearance kinetics, and vascular circulation half-life in the large animal model. Further study of CF800 in the porcine model will provide the blueprints to translating nano-liposome to the clinical setting.

## Supporting information

S1 Text Individual surveyee response on potential clinical application of CF800. Contains the individual responses to the survey questions. Table A. Surveyee response on smallest discernable pulmonary vessel branch generation in image. Table B. Surveyee response on comfort level in performing a lobectomy with the images (scale 1–10).
(DOCX)
