# Supplementary material for: Nanoparticle-based CT visualization of pulmonary vasculature for minimally-invasive thoracic surgery planning
Source: PLoS One. 2019 Jan 17;14(1):e0209501. doi: 10.1371/journal.pone.0209501 (PMC6336249; doi:10.1371/journal.pone.0209501)
Supplement: S1 Text — Contains the individual responses to the survey questions. Table A. Surveyee response on smallest discernable pulmonary vessel branch generation in image. Table B. Surveyee response on comfort level in performing a lobectomy with the images (scale 1–10). (DOCX) [file pone.0209501.s001.docx]

# **Supporting Information**

**Individual surveyee response on potential clinical application of CF800**

**Table A. Surveyee response on smallest discernable pulmonary vessel branch generation in image**

| **Surveyee** | **Rabbit (Pre-inj)** | **Rabbit (Post-inj)** | **Pig  (Pre-inj)** | **Pig  (Post-inj)** |
| --- | --- | --- | --- | --- |
| **1** | 7 | 9 | 8 | 8 |
| **2** | 4 | 6 | 8 | 9 |
| **3** | 4 | 6 | 4 | 5 |
| **4** | 5 | 6 | 4 | 6 |
| **5** | 9 | 9 | 5 | 4 |
| **6** | 6 | 7 | 4 | 4 |
| **7** | 3 | 4 | 4 | 4 |
| **MEAN** | **5.43** | **6.71** | **5.29** | **5.71** |

**Table B. Surveyee response on comfort level in performing a lobectomy with the images (scale 1-10)**

| **Surveyee** | **Rabbit (Pre-inj)** | **Rabbit (Post-inj)** | **Pig  (Pre-inj)** | **Pig  (Post-inj)** |
| --- | --- | --- | --- | --- |
| **1** | 8 | 9 | 9 | 9 |
| **2** | 3 | 7 | 3 | 5 |
| **3** | 5 | 7 | 5 | 7 |
| **4** | 4 | 8 | 5 | 10 |
| **5** | 7 | 10 | 5 | 6 |
| **6** | 2 | 8 | 2 | 6 |
| **7** | 4 | 8 | 5 | 10 |
| **MEAN** | **4.71** | **8.14** | **4.86** | **7.57** |
